# Black Lives Matter Worldwide: Retooling Precision Oncology for True Equity of Cancer Care

**DOI:** 10.1016/j.xcrm.2020.100079

**Published:** 2020-08-25

**Authors:** Padma Sheila Rajagopal, Olufunmilayo I. Olopade

**Affiliations:** 1 Section of Hematology/Oncology, Department of Medicine, University of Chicago Medical Center, Chicago, IL 60637, USA; 2 Center for Clinical Cancer Genetics and Global Health, University of Chicago Medical Center, Chicago, IL 60637, USA

## Abstract

In a recent issue of *Cancer Cell*, Carrot-Zhang et al. identified ancestry-specific molecular variants and expression changes among patients from The Cancer Genome Atlas (TCGA).^1^ Their study findings and limitations highlight the critical need to diversify populations represented in cancer genomics research.

## Main Text

Ever since the “Philadelphia chromosome” was characterized as a leukemia-defining translocation, cancer has been considered a genetic disease. Molecular alterations can now inform diagnosis, treatment targets, and predictive and prognostic biomarkers across cancer types. Efforts such as The International Hapmap Project, the 1000 Genomes Project, and the Trans-Omics for Precision Medicine (TOPMed) Program have all emphasized the need to include participants from diverse international backgrounds, yet the vast trove of genomic and transcriptomic data in cancer genomics research continues to overwhelmingly represent patients of European ancestry. Of the major genomic datasets in oncology that offer publicly available ancestry information, The Cancer Genome Atlas (TCGA) includes 83% European ancestry patients compared to 6% each of African and East Asian patients,^2^ while the Pan-Cancer Analysis of Whole Genomes (PCAWG) includes 77% European ancestry patients compared to 16% East Asian and 5% African patients.^3^

Prior work on genomic associations between ancestry and cancer has focused largely on the direct relationship between genomic alterations and disparities in outcomes. For example, patients of African ancestry with cancer have worse overall survival in spite of similar or decreased incidence rates relative to patients of European ancestry.^4^ In breast cancer, while socioeconomic and cultural factors explain some of this disparity, women of African ancestry also suffer higher rates of the more aggressive triple-negative or HER2-positive subtypes, with at least 40% of this subtype difference estimated as genetically driven.^5^ However, it remains unknown why breast cancer subtypes show ancestry-specific differences as well as what biological factors drive evolving tumors toward a given subtype.^6^

Carrot-Zhang and colleagues are among only a handful of groups asking how genetic ancestry may influence cancer development. In their recently published article, they examined associations between ancestry (defined via population-specific genomic markers) and somatic (cancer-only) alterations, DNA methylation sites, messenger RNA (mRNA) expression, and microRNA (miRNA) expression across the 10,678 patients and 33 cancer types represented in TCGA. Their study expanded on prior work to include epigenetic and post-transcriptional regulation as well.^1^ In addition, they performed integrated analyses of these data types to study ancestry-specific expression quantitative trait loci (eQTLs) and cancer immunogenicity. They now demonstrate that across cancer genomics research, ancestry must be accounted for in experimental design.

Ignoring differential distributions of cancer-specific subtypes among patients of differing ancestral populations (for example, the higher prevalence of triple-negative subtype among African ancestry patients with breast cancer) introduces confounding, thus imperiling the translational significance of research findings. The Cancer Genetic Ancestry Atlas developed by Yuan et al. previously associated *PIK3CA* mutations with European ancestry and *TP53* mutations with African ancestry.^2^ These relationships are now argued to be subtype specific, a pattern validated by studying breast tumors from patients in Nigeria as well as those from TCGA European and African ancestry patients.^5^^,^^7^ The interaction between *TP53* mutations in patients of African ancestry and subtypes also associates with higher risk of recurrence.^5^

Also importantly, with their work on methylation and microRNAs, Carrot-Zhang and colleagues raise the larger question of how genes literally interact with geography and environment to influence cancer development. The group consistently saw more significant methylation sites and greater consistency in methylation when examining ancestry within specific cancer types compared to a pan-cancer analysis. Similarly, the largest effect sizes for ancestry-specific differences in miRNA expression were within specific cancer types. DNA methylation markers in cancer have been previously shown to perform well in differentiating geographic origins.^8^ Alterations in miRNAs expression, among many other functions, correlate with environmental exposures, illuminating a potential mechanism bridging ancestral genes and clinical cancer development that must be considered in further detail.^9^

The limitation consistently highlighted by Carrot-Zhang et al. across these interesting findings is power. To find genomic variants specific to African ancestry, the authors estimate 50% power to detect an association with a given cancer for a minimum odds ratio of 1.5, assuming a mutation frequency of 0.3. These standards will only detect the most obvious ancestry-specific associations. If we were to design an experiment to seek variants with ancestry-specific cancer associations at 80% power and a type-I error of 0.05, we would ideally need more than 15,000 patients of African ancestry to participate in research. The sum total of patients of African ancestry in TCGA is 651. That estimate does not even fully account for well-known analytic issues in genomics such as shorter linkage disequilibrium in patients of African ancestry (necessitating whole-genome sequencing to find disease-associated variants) and lack of full representation of African-specific variants in the reference human genome.^10^ Given this paucity of data on minority populations in the U.S., efforts such as the Polyethnic 1000 from the New York Genome Center highlight how to move toward improving annotated data on non-European ancestry cancer genomes.

In the United States, we are now being asked to face the inequity in our healthcare system head-on, driven by the concurrent pandemics of systemic racism and COVID-19. Vulnerable populations face significant potential harm both from the growing global burden of cancer and the fact that the majority of individuals currently diagnosed with cancer will not benefit from either precision oncology-based preventative risk assessment or targeted therapies. Scientific collaborations and mutually beneficial international partnerships at intersection of patient care and research can be leveraged to actively engage diverse populations of patients in genetics research across racial, geographic, and economic barriers. Now more than at any time in history, we have the responsibility and capacity not only to ask how we can best serve *every* cancer patient in the world using genomic research, but also to use science and technology to promote equity.
